# Effects of Active Dry Yeast Supplementation in In Vitro and In Vivo Nutrient Digestibility, Rumen Fermentation, and Bacterial Community

**DOI:** 10.3390/ani14192916

**Published:** 2024-10-09

**Authors:** Haitao Liu, Fei Li, Zhiyuan Ma, Miaomiao Ma, Emilio Ungerfeld, Zhian Zhang, Xiuxiu Weng, Baocang Liu, Xiaoyu Deng, Liqing Guo

**Affiliations:** 1 State Key Laboratory of Herbage Improvement and Grassland Agro-Ecosystems, Lanzhou University, Lanzhou 730020, China; liuht19@lzu.edu.cn (H.L.); lfei@lzu.edu.cn (F.L.); zhangzha20@lzu.edu.cn (Z.Z.); wengxx@lzu.edu.cn (X.W.); 2 College of Pastoral Agriculture Science and Technology, Lanzhou University, Lanzhou 730020, China; 3 Key Laboratory of Grassland Livestock Industry Innovation, Ministry of Agriculture and Rural Affairs, Lanzhou 730020, China; 4 Animal Husbandry Work Station of Ningxia, Yingchuan 750002, China; nannontonban@126.com; 5 Centro Regional de Investigación Carillanca, Instituto de Investigaciones Agropecuarias INIA, Vilcún 4880000, Chile; emilio.ungerfeld@inia.cl; 6 Aksu Tycoon Feed Co., Ltd., Aksu 843000, China; baocangliu@outlook.com (B.L.); tkxiaoyu.deng@outlook.com (X.D.); 7 Tecon Pharmaceutical Co., Ltd., Suzhou 215000, China; glqaibjl@163.com

**Keywords:** active dry yeast, digestibility, fermentation, rumen

## Abstract

**Simple Summary:**

This study evaluated the influence of four active dry yeasts (ADYs) on nutrient digestibility and rumen fermentation in lambs through in vitro and in vivo experiments. Notably, Vistacell (Vis) and Procreatin7 (Pro) showed enhanced total gas production and pH levels in vitro. Vis improved in vivo propionate molar proportion, NDF digestibility, and total VFA concentration, suggesting it is the most suitable for lamb growth. However, the study highlighted discrepancies between in vitro and in vivo outcomes, cautioning against directly translating batch culture results to live animal effects.

**Abstract:**

This study assessed the impact of active dry yeast (ADY) on nutrient digestibility and rumen fermentation, using both in vitro and in vivo experiments with lambs. In vitro, ADYs were incubated with rumen fluid and a substrate mixture to assess gas production, pH, volatile fatty acid (VFA) profiles, and lactate concentration. In vivo, Hu lambs were randomly assigned to five dietary treatments: a control group and four groups receiving one of two dosages of either Vistacell or Procreatin7. Growth performance, nutrient digestibility, rumen fermentation parameters, and bacterial community composition were measured. Pro enhanced the propionate molar proportion while it decreased the n-butyrate molar proportion. Vis reduced the lactate concentration in vitro. In the in vivo experiment, Vis increased the propionate molar proportion and the Succinivibrionaceae_UCG-001 abundance while it decreased the n-butyrate molar proportion and the Lachnospiraceae_ND3007 abundance. Additionally, Vis showed a greater impact on improving the NDF digestibility and total VFA concentration in vivo compared to Pro. Overall, the effects of ADYs on rumen fermentation were found to vary depending on the specific ADY used, with Vis being the most suitable for lamb growth. It was observed that Vis promoted propionate fermentation and Succinivibrionaceae_UCG-001 abundance at the expense of reduced n-butyrate fermentation and Lachnospiraceae_ND3007 abundance. Importantly, differences were noted between the outcomes of the in vitro and in vivo experiments concerning the effects of ADYs on rumen fermentation, highlighting the need for caution when generalizing batch culture results to the in vivo effects of ADYs.

## 1. Introduction

The practice of feeding high concentrate diets to finishing feedlot lambs increases the growth rate and improves the feed efficiency but also increases the risk of metabolic disorders such as acidosis [1], which is attributed to the accumulation of VFA and/or lactate, resulting in a decreased rumen pH, negatively affecting fibrolytic microorganisms [2]. A low rumen pH and an increased rumen passage rate resulting from high concentrate feeding can also reduce rumen fiber digestion [3]. Hence, it is important to alleviate these negative effects of high concentrate diets to ensure healthy and efficient ruminant production.

The active dry yeast (ADY) *Saccharomyces cerevisae*, resulting from the brewery or baking industries, is used in the ruminant production industry as a probiotic feed additive [4]. High concentrate diets typically contain low levels of fiber, which can lead to a reduction in the fibrolytic population in the rumen [5]. Supplementing ADY to high concentrate diets of ruminants can stabilize the rumen pH and thus promote rumen fibrolytic activity [6]. Some studies have indicated that ADY can survive in the rumen and potentially interact with rumen bacteria to promote the growth of fibrolytic species [4,7]. Supplementing ADY has been proposed as a strategy to support rumen fibrolytic bacteria and enhance fiber digestion efficiency. This is of great interest in ruminants subjected to high concentrate diets. Further studies are needed to provide a more comprehensive understanding of the specific effects of ADY on rumen bacterial populations as well as the mechanisms through which ADY interacts with rumen bacteria to support fiber digestion and mitigate the negative impacts of high concentrate feeding on rumen health and function.

The present study hypothesized that the supplementation of specific ADY products in high concentrate diets fed to finishing lambs could modulate the rumen bacterial population towards a more favorable profile characterized by an increase in fibrolytic bacteria. We assessed the impact of ADY supplementation on fiber degradation, volatile fatty acid production, and the overall nutrient utilization in the rumen, aiming to improve rumen health and performance in ruminants on high concentrate diets.

## 2. Materials and Methods

All the animals and procedures were approved by the Animal Care and Use Committee of Lanzhou University, Lanzhou, China (approval number CPAST-2020–LI-3).

### 2.1. In Vitro Experiment

The in vitro fermentation experiment consisted of a control group without any ADY treatment and eight treatments with one of four commercial ADY sources at two dosages each. The four ADY sources included FB (≥2.0 × 10^8^ CFU/g, Angel Yeast Co., Ltd., Yichang, China), Procreatin7 (≥2.0 × 10^8^ CFU/g, Guangxi Danbaoli Yeast Co., Ltd., Laibin, China), Vistacell (≥2.0 × 10^8^ CFU/g, AB Vista, Wiltshire, UK), and YSF (≥1.0 × 10^8^ CFU/g, Lesaffre Industries Co., Ltd., Paris, France), with all the information of the CFU per gram provided by the manufacturers. The two doses of each product targeted 6 × 10^5^ and 1.2 × 10^6^ CFU/mL of fermentation fluid and were calculated considering the minimum guaranteed CFU/g informed by each manufacturer. From this point on, the four treatments that supplemented each yeast in vitro and in vivo will be referred to as FB, Pro, Vis, and YSF.

The detail of the in vitro incubation process is described in our published paper [8]. In brief, the in vitro fermentation flasks contained 0.9 g of substrate, 30 mL of rumen fluid, 60 mL of CO_2_-saturated artificial saliva [9], and one ADY at one of the two dosages. The substrate incubated consisted of 25% corn silage, 15% alfalfa, 25% corn grain, 15% corn bran, 11% soybean meal, 8% cottonseed meal, and 1% limestone and contained 30.6% NDF, 24.0% starch, and 16.3% CP on a DM basis. Rumen contents were obtained from three rumen-cannulated male *Hu* lambs that were fed a commercial diet (Gansu Runmu Biotechnology Co., Ltd., Jinchang, China) containing 39.2% NDF and 13.4% CP before the morning feeding. Rumen fluids were obtained by straining rumen contents through four layers of cheesecloth and were taken to the laboratory for mixing with medium and inoculating fermentation flasks within 30 min. All the laboratory procedures involving the processing and inoculation of rumen fluid were conducted under CO_2_ protection.

Each treatment, including the control, had six fermentation flasks with two flasks inoculated with each animal’s rumen fluid, resulting in three biological replicates, each in turn containing two technical replicates. Before adding them to the fermentation flasks, the ADYs were suspended in ddH_2_O to target a concentration of 10^8^ CFU/mL according to the information of the minimum CFU per gram provided by the manufacturers. This resulted in 0.36 and 0.72 mL of ADY suspension delivered to flasks with targeted doses of 6 × 10^5^ and 12 × 10^5^ CUF/mL of fermentation broth, respectively. The volume of added water delivered in the ADY suspension received by the different treatments was not equalized by adding ddH_2_O. The flasks were incubated at 39 °C for 24 h in a fermentation system with automatic exhaust recording exhaust gas volume (AGRS3, Beijing Boxiang Xingwang Technology Co., Ltd., Beijing, China).

After 24 h of incubation, the fermentation flasks were placed on ice to arrest the microbial activity. The bottles were opened, and the pH was measured using a portable pH meter (PHB4, Shanghai INESA & Scientific Instrument Co., Ltd., Shanghai, China). A 10-mL sample of fermentation liquid mixed with 2 mL meta-phosphoric acid (25%, g/mL) and a 5-mL sample of fermentation liquid was stored at −20 °C for subsequent analysis of VFA and lactic acid, respectively.

### 2.2. Animal Trial

Vistacell and Procreatin7 were selected for the animal trial because of their in vitro effects on pH stabilization and a decrease in lactic acid concentration. The animal trial consisted of a control treatment without any yeast supplementation and four treatments with one of Vistacell or Procreatin7 supplemented at 0.60 or 1.20 g/d, which are dosages that are recommended for non-lactating and lactating dairy cows, respectively [4]. A total of thirty healthy 4-month-old Hu lambs with a similar initial body mass (36.5 ± 3.22 kg, mean ± SD) were randomly allocated to one of the five dietary treatments. The basal diet was provided as a pelleted TMR consisting of 20% barley straw, 28% corn, 27% corn gluten feed, 14% corn germ meal, 5% cottonseed meal, 4% molasses, 1% CaCO_3_, 0.5% NaCl, and 0.5% vitamin and trace mineral premix. The basal diet contained 31.1% NDF, 24.4% starch, and 13.7% CP (DM basis). One kilogram of premix included Fe 25 mg, Mn 40 mg, Zn 40 mg, Cu 8 mg, I 0.3 mg, Se 0.2 mg, Co 0.1 mg, vitamin A 940 IU, vitamin D 111 IU, and vitamin E 20 IU. The lambs were raised in individual pens (1.8 m × 1.25 m × 1 m) and were ad libitum fed at 8:00 and 18:00 daily to allow 15% refusals. Each ADY additive was mixed with 100 g of ground diet and was provided entirely before the morning feeding to ensure the animals ate all the provided ADY. Water was available at all times via an automatic water supply system.

Following fifteen days of feeding, the lambs experienced five days of consecutive fecal and urine collection, followed by a day of rumen fluid collection. Feces were collected in bags attached to the animals in a way to avoid urine contamination. The urine was collected in a bucket through a funnel-shaped bottom pan located underneath the pen. A 10% sample of the daily fecal output of each lamb was sampled, and samples of the 5-day fecal sampling period were pooled by animal. For the determination of total N in the urine, 20% of the daily urine was sampled and pooled according to the animal. Ten milliliters of 10%(g/mL) sulfuric acid were added to the fecal and urine samples used for N determination to avoid ammonia volatilization.

The rumen contents were collected through oral tubes before and 3 h after the morning feeding, with the earliest collected rumen fluid discarded to rule out saliva contamination. The pH of the rumen fluid was measured immediately using a portable pH meter (PHB4, Shanghai INESA & Scientific Instrument Co., Ltd., Shanghai, China). We took 9 mL of rumen fluid and added 1 mL of 25% (g/mL) meta-phosphoric acid, mixed it, centrifuged it at 2000× *g* for 10 min, and froze the supernatant at −20 °C for subsequent analysis of the VFA concentration. Five-milliliter samples of rumen fluid were snap-frozen in liquid nitrogen and stored at −80 °C for subsequent analysis of the bacterial community composition.

### 2.3. Analytical

The plate counting method was used to determine the active yeast count in CFU [10]. In short, 10× serial diluted yeast suspensions were spread onto Petri dishes containing YPD solid medium (1% yeast extract, 2% peptone, 2% glucose, 2% agar, 93% ddH_2_O). Then, the colonies of viable yeast cells were counted in each plate after incubation at 38 °C for 24 h.

Proximate analyses were conducted according to AOAC [11]. In brief, the CP was determined using an automatic Kieldahl apparatus (Shanghai Shengsheng Automation Analysis Instrument Co., Ltd., Shanghai, China); the NDF and ADF were measured using the Van Soest method [12] with a fiber analyzer (ANKOM A200i, ANKOM Technology, Macedon, NY, USA); the gross energy was measured using a calorimeter (C3000, IKA Laboratory Technology, Staufen, Germany).

The ammonia concentration in the rumen fluid was measured using the phenol-hypochlorite method [13]. Individual VFA concentrations were measured using a gas chromatograph (TRACE1300, Thermo Scientific, Milan, Italy). The lactic acid concentration was measured using a 3-(4,5-dimethylthiazol-2-yl)-2,5-diphenyl tetrazolium bromide (MTT) colorimetric method [14] with a commercial kit (Nanjing Jiancheng Bioengineering Institute, Nanjing, China).

Rumen fluids sampled at the two time points were pooled by animal for the analysis of the bacterial community composition. A sand-beating method [15] was performed to extract the microbial DNA in the rumen fluid. Bacterial V3-V4 amplicon sequencing was performed by Biozeron Corp., Wuhan, China, with primers of 341F (5′-CCTACGGGAGGCAGCAG-3′) and 806R (5′-GGACTACHVGGGTWTCTAAT-3′) [16].

The subsequent bioinformatic analysis based on zero-radius OTU (ZOTU) is described in detail in our previous paper [15]. In brief, ZOTU was generated by the denoise3 algorithm after quality control using usearch v5 [17]. Taxonomic annotation was performed by mothur v4 [18] against silva.nr v138 [19]. The ZOTU table was created by mapping the raw sequences to ZOTU sequences using vsearch v2 [20]. Alpha diversity and principal coordinate analysis (PcoA) were performed using an internal R script (https://github.com/sleepvet/MicrobialDiversity, accessed on 1 March 2023).

### 2.4. Statistical Analyses

The response variables, besides the fermentation parameters assessed in vivo, were measured only at a single time point and fitted to a mixed linear model: Response = μ + T_i_ + Animal_j_ + error_ij_, where T is a fixed effect of treatment (including control) and animal is a random effect. The response variables sampled at more than one time were fitted to a mixed linear model: Response = μ + T_i_ + Sampling time_j_ + T × Sampling time_ij_ + Animal_k_ + error_ijk_, where T and sampling time are fixed effects, animal is a random effect, and sampling time was also set as a repeated measurement with an AR(1) covariance structure. When the interaction between the sampling time and treatment was significant, a reduced model was fitted for each sampling time point.

Pre-planned contrasts were used to evaluate the average of each ADY supplementation against the control treatment: Contrast (ADY vs. Con) = Control − 0.5 × (ADY_dose0.6_ + ADY_dose1.2_). If the contrast of a particular ADY resulted as significant (*p* < 0.05), polynomial contrasts of the dosage effect of the ADY were evaluated. Pre-planned contrasts were also used for pairwise comparison of the average of both ADY products: Contrast (ADY1 vs. ADY2) = (ADY1_dose0.6_ + ADY1_dose1.2_) − (ADY2_dose0.6_ + ADY2_dose1.2_).

All the statistics were performed in R v4.2 [21]. The linear mixed model was completed using lme module in the nlme package (https://svn.r-project.org/R-packages/trunk/nlme, accessed on 12 May 2022). The pre-planned contrast and polynomial contrast were conducted using the standardize package (https://github.com/CDEager/standardize, accessed on 12 May 2022). The relative abundance data of the bacterial community was log-transformed to meet the requirements of data distribution normalization.

## 3. Results

After plate culture counting, we found that the live yeast cells of FB, Pro, Vis, and YSF were 2.1 × 10^8^, 1.5 × 10^8^, 2.2 × 10^8^, and 1.4 × 10^8^ CFU/g, respectively.

### 3.1. In Vitro Experiment

The total gas volume increased at a high dose of Pro and Vis (*p* ≤ 0.04, Table 1). Among the ADY sources, Pro produced (*p* = 0.05), and Vis tended to produce (*p* = 0.10), more total gas than YSF and FB. Pro and Vis increased or tended to increase the pH (*p* ≤ 0.09). No differences among the ADYs were observed (*p* ≥ 0.12). Vis increased the total VFA concentration (quadratic effect; *p* = 0.01), while other ADYs did not have any effect (*p* ≥ 0.65). None of the ADYs affected the molar percentage of acetate (*p* ≥ 0.51). The molar proportion of propionate was increased by Pro (quadratic effect; *p* < 0.001) and tended to increase with YSF (*p* = 0.07). A pairwise comparison of the ADY sources did not reveal any differences in the molar percentage of propionate (*p* ≥ 0.15). Pro decreased (*p* = 0.009), and FB and YSF tended to decrease (*p* ≤ 0.10), the molar percentage of n-butyrate. The molar percentage of n-butyrate was lower with Pro than with Vis (*p* = 0.04). The acetate to propionate molar ratio was decreased by Pro (*p* = 0.002) and tended to be decreased by YSF (*p* = 0.08). Pro had a lower acetate-to-propionate molar ratio than the other three ADYs (*p* ≤ 0.003). The lactate concentration was decreased by Vis only (*p* = 0.04).

### 3.2. In Vivo Experiment

Neither Pro nor Vis affected BM and DMI (*p* ≥ 0.46, Table 2). The digestibility of DM, OM, and CP was not affected by Pro or Vis (*p* ≥ 0.21). The digestibility of NDF and ADF was not affected by Pro (*p* ≥ 0.40) while Vis increased the NDF and ADF digestibility (quadratic effect; *p* = 0.02). No N balance response was affected by Pro or Vis (*p* ≥ 0.12; Table 3).

There were no treatment interactions for the sampling time for any rumen fermentation variable (*p* ≥ 0.18, Table 4). Pro supplementation did not affect the TVFA concentration (*p* = 0.49) while Vis supplementation tended to increase the TVFA concentration (*p* = 0.08). However, there was no difference in the effect of Pro and Vis supplementation on TVFA (*p* = 0.19). Neither Pro nor Vis supplementation affected the rumen pH (*p* ≥ 0.16). Supplementation with Pro did not affect the acetate molar percentage (*p* = 0.87) while Vis supplementation tended to decrease the acetate molar percentage (*p* = 0.08), resulting in a lower acetate molar percentage than Pro (*p* = 0.02). Vis increased (*p* = 0.006), and Pro tended to increase, the propionate molar percentage (*p* = 0.09), with no differences between Pro and Vis (*p* = 0.12). Vis decreased (*p* = 0.02), and Pro tended to decrease, the n-butyrate molar percentage (*p* = 0.06), with no differences between Pro and Vis (*p* = 0.48). Neither the Pro nor Vis treatments affected the acetate-to-propionate ratio (*p* = 0.50).

**Figure 1:**
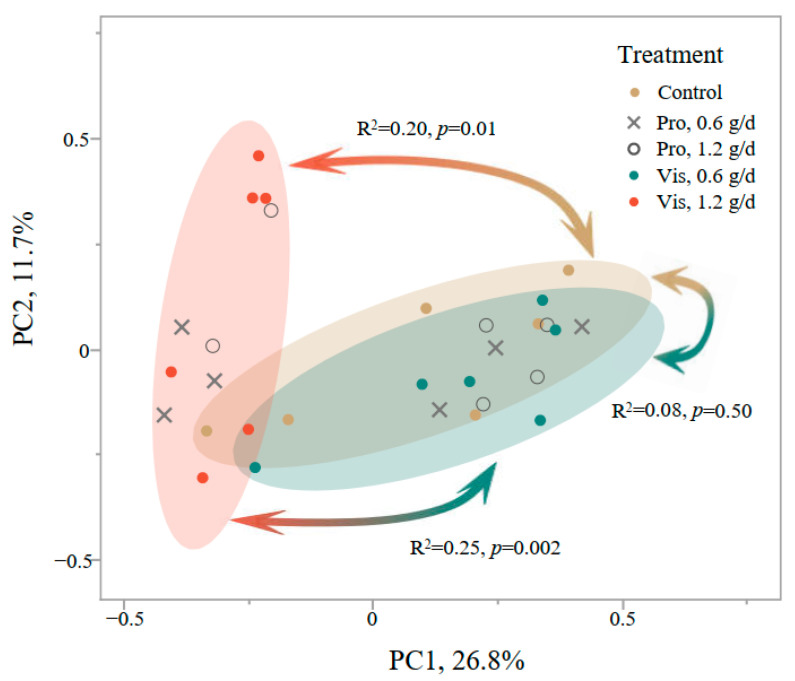
Principal coordinate analysis of active dry yeast (ADY) supplementation on rumen bacterial community based on Bray–Curtis dissimilarity matrix at ZOTU level. The ADY sources included Procreatin7 (Pro, Guangxi Danbaoli Yeast Co., Ltd., Laibin, China) and Vistacell (Vis, AB Vista, Wiltshire, UK). The control treatment did not contain any ADY. In particular, the three supplementation level pairs of Vis were compared using the adonis module in the vegan package.

The PcoA showed a separation between Vis supplementation at 1.2 g/d and the control treatment (*p* = 0.01, Figure 1) while Vis supplementation at 0.6 g/d did not differ from the control (*p* = 0.50). Both levels of Pro supplementation showed no difference from the control (*p* ≥ 0.23).

The supplementation of Pro had no influence on the observed ZOUT and PD_whole_tree (*p* ≥ 0.83, Table 5) but quadratically decreased the Pielou index (*p* = 0.04). The supplementation of Vis linearly decreased the Pielou index (*p* = 0.02). There were no differences between the Pro and Vis treatments on the alpha diversity indices of the rumen bacterial community (*p* ≥ 0.47).

We detected a total of 150 bacterial genera using 16S rRNA gene amplicon sequencing, with the top 20 genera accounting for 89.3% of the total bacteria, making them the predominant bacteria in the rumen (Table 6). The supplementation with Pro or Vis did not affect the abundance of *Prevotella*, *Succinivibrio*, Prevotellaceae_UCG-001, *Selenomonas*, Prevotellaceae_unclassified, Rikenellaceae_RC9, *Succiniclasticum*, *Fibrobacter*, Muribaculaceae_ge, Prevotellaceae_YAB2003, *Ruminococcus*, Lachnospiraceae_unclassified, and *Oribacterium* (*p* ≥ 0.11). Succinivibrionaceae_UCG-001, Selenomonadaceae_unclassified, Clostridia_UCG-014, F082_ge, Prevotellaceae_UCG-003, and *Treponema* were not influenced by Pro supplementation (*p* ≥ 0.14), but Vis increased Succinivibrionaceae_UCG-001 (*p* = 0.04) and promoted a quadratic response in (*p* = 0.05). Clostridia_UCG-014 had a tendency to increase with Vis supplementation (*p* = 0.10), and the abundance of F082_ge, Prevotellaceae_UCG-003, and *Treponema* linearly decreased with an increasing Vis supplementation level (*p* ≤ 0.04).

## 4. Discussion

The TVFA are the main metabolic products of carbohydrate fermentation by rumen microbes [22]. Increased TVFA production reflects the extent of substrate fermentation by microbes, representing rumen fermentation activity [22]. Total gas production is also an important indicator to evaluate the activity of microbes in in vitro fermentation [23]. The results from the in vitro experiment show that only Pro and Vis enhanced the total gas production, with Vis also promoting an increase in the TVFA concentration. These findings suggest that these two additives play a role in promoting rumen fermentation. Pro and YSF increased the molar percentage of propionate to varying degrees in vitro. Similar to our results, in vitro fermentation inoculated with rumen fluid of dairy cows showed an increase in the molar percentage of propionate by ADYs [24]. In most cases, acetate is the main product of yeast fermentation, not propionate [25]. Increases in the propionate concentration as a consequence of yeast supplementation have been attributed to the substances produced by yeast metabolism promoting propionate producers in the rumen [4,24]. Propionate production incorporates metabolic hydrogen. Our results suggest that the ADY may have redirected metabolic hydrogen towards propionate production. Additionally, Vis resulted in a decreased lactate concentration. Because of these results, we selected Vis and Pro for their evaluation in vivo.

The meta-analysis by Sales [26] showed that the effects of ADY on nutrient digestibility vary substantially across studies, especially for fiber digestibility. This is consistent with our finding that Vis but not Pro improved fiber digestibility in vivo. Similar to our results, McGinn et al. [27] also found that Pro had little effect on the NDF digestibility of steers. While we verified Vis to enhance fiber digestion in finishing feedlot lambs, it had no effect on fiber digestibility in lactating dairy cows [28]. Sales [26] further noted that ADY had a lesser effect on dietary NDF digestibility under high concentrate diet conditions, which was primarily attributed to the limited action of yeast in the rumen due to the rapid rumen passage rate induced by a high concentrate diet. Even though the diets offered to lambs in our study were relatively high in concentrate content, favorable effects of Vis on fiber digestion were noted. It has been reported that the supplementation of ADY can decrease the fecal N excretion of lambs [29]. However, our results align with others that found no effect of ADY on the N balance in finishing lambs [30,31].

Vis but not Pro supplementation tended to increase the TVFA concentration in the rumen. This finding confirms that Vis has a greater impact on rumen fermentation than Pro. In vivo, as opposed to in vitro, Vis shifted rumen fermentation from n-butyrate to propionate to a greater extent than Pro. Conflicting in vitro and in vivo results have been discussed [32], underscoring the need for caution when only in vitro results are available. Additionally, Garcia Diaz et al. [33] did not observe the effect of Pro on the sheep rumen VFA profile in vivo. While there have been no reports about the effects of Vis in rumen fermentation in lambs, Vis has been reported not to affect the rumen VFA profile in lactating dairy cows [28] and finishing feedlot steers [34].

Lactate is produced in the rumen as an intermediate product of carbohydrate fermentation, which can accumulate with highly fermentable diets [35]. Studies by Lynch and Martin [36] and Lila et al. [24] found that ADY supplementation increased propionate production and reduced lactate accumulation. The authors suggested that ADY may promote lactate utilization by stimulating the growth of lactate-utilizing bacteria [24,36]. This is important because excessive lactate accumulation can lead to rumen acidosis, which can have negative impacts on animal health and productivity [35]. Malekkhahi et al. [37] related ADY to the metabolism of lactate to propionate and n-butyrate in the rumen of lactating dairy cows by *Megasphaera*. Even though the lactate concentration was decreased by Vis in our in vitro experiment, the lactate basal concentration in the control treatment was rather small, and, clearly, decreases in lactate of about 25 µ*M* would have been unnoticeable if lactate had been metabolized to propionate and butyrate, which were at concentrations close to three orders of magnitude higher than lactate. Moreover, *Megasphaera* spp. were not detected in the major bacteria, which agrees with our presumption that lactate metabolism was quantitatively unimportant in our in vitro experiment.

The bacterial alpha diversity and beta diversity were influenced by Vis but not by Pro. This observation aligns with our findings of Vis affecting the NDF digestibility and rumen VFA profile in vivo. Pro supplementation decreased the abundance of the putative genus Selenomonadaceae_unclassified, which is presently functional information. A recent in vitro analysis indicated that the abundance of Selenomonadaceae_unclassified increased in association with a decrease in the propionate molar percentage [38]. In agreement, we observed that Pro supplementation decreased the abundance of Selenomonadaceae_unclassified and the propionate molar percentage. The relative abundances of F082_ge, Prevotellaceae_UCG-003, and Lachnospiraceae_ND3007 were decreased by Vis supplementation. F082_ge and Prevotellaceae_UCG-003 are associated with the production of propionate using the succinate pathway [39,40]. In contrast, in our study, Vis supplementation decreased the relative abundance of F082_ge and Prevotellaceae_UCG-003 but increased the molar percentage of propionate in the rumen. This indicates that other bacteria or pathways may be compensating for the decrease in the F082_ge and Prevotellaceae_UCG-003 abundance to shift the fermentation profile towards propionate. Lachnospiraceae_ND3007 produces n-butyrate from the degradation of pectins [41]. It is possible that the decreased abundance of Lachnospiraceae_ND3007 resulting from Vis supplementation contributed to account for the decrease in the molar percentage of n-butyrate in the rumen of lambs, although the basal abundance of Lachnospiraceae_ND3007 was quite low and it is, therefore, uncertain how much Lachnospiraceae_ND3007 might have contributed to n-butyrate production.

## 5. Conclusions

The study found that the impact of ADYs on rumen fermentation varies, with Vis yeast being optimal for lamb growth. It enhanced propionate and Succinivibrionaceae_UCG-001 while reducing n-butyrate and Lachnospiraceae_ND3007. The in vitro and in vivo results diverged, cautioning against generalizing batch culture findings.

## Figures and Tables

**Table 1:** Effects of active dry yeast (ADY) on in vitro fermentation parameters (*n* = 3).

Item	Total Gas, mL	pH	TVFA, m*M*	Molar Percentage of Individual VFA, %				Ace/Pro	Lactate, μ*M*
				Acetate	Propionate	n-Butyrate	Others ^2^		
Treatment, 10^5^ CFU ADY ^1^/mL fermentation broth									
Control, 0	308	5.87	113	58.1	18.3	18.2	5.29	3.16	98.1
FB, 6	306	6.02	108	58.4	17.8	17.8	5.79	3.27	112
FB, 12	329	6.08	111	58.6	20.8	15.3	5.12	2.81	82.5
Pro,6	307	5.96	106	57.7	21.1	15.7	5.37	2.74	113
Pro, 12	338	6.11	126	57.7	21.8	15.1	5.12	2.64	91.8
Vis, 6	294	5.90	111	58.1	17.8	18.2	5.82	3.26	96.0
Vis, 12	335	6.08	135	58.7	20.0	15.9	5.27	2.93	73.8
YSF, 6	297	5.90	108	58.6	20.3	15.5	5.27	2.89	98.2
YSF, 12	290	5.98	112	58.3	19.1	16.6	5.77	3.04	117
SEM	10.1	0.07	6.5	0.54	0.67	0.73	0.176	0.097	9.10
*p*-value									
FB vs. Con	0.29	0.04 ^Q^	0.65	0.52	0.20	0.10	0.44	0.24	0.95
Pro vs. Con	0.04 ^Q^	0.08	0.74	0.58	<0.001 ^Q^	0.009 ^L^	0.85	0.002 ^L^	0.73
Vis vs. Con	0.03 ^Q^	0.09	0.01 ^Q^	0.58	0.19	0.23	0.25	0.55	0.04 ^Q^
YSF vs. Con	0.87	0.14	0.69	0.51	0.07	0.06	0.29	0.08	0.75
FB vs. Pro	0.71	0.35	0.65	0.15	0.001	0.13	0.24	<0.001	0.41
FB vs. Vis	0.82	0.26	0.05	0.89	0.47	0.58	0.64	0.47	0.05
FB vs. YSF	0.11	0.12	0.94	0.99	0.45	0.50	0.72	0.37	0.20
Pro vs. Vis	0.57	0.90	0.29	0.19	<0.001	0.04	0.11	<0.001	0.04
Pro vs. YSF	0.05	0.52	0.38	0.15	0.009	0.39	0.14	0.003	0.59
Vis vs. YSF	0.10	0.60	0.06	0.88	0.15	0.22	0.90	0.11	0.03

^1^: The four ADY sources included FB (Angel Yeast Co., Ltd., Yichang, China), Procreatin7 (Pro, Guangxi Danbaoli Yeast Co., Ltd., Laibin, China), Vistacell (Vis, AB Vista, Wiltshire, UK), and YSF (Lesaffre Industries Co., Ltd., Paris, France). The control treatment did not contain any ADY. ^2^: Others included n-valerate, iso-butyrate, and iso-valerate. ^L,Q^: Polynomial contrasts for dosage effects (linear, L; quadratic, Q) of individual ADYs.

**Table 2:** Effects of active dry yeast (ADY) on growth performance and nutrient digestibility of Hu lambs (*n* = 6).

Item	BM, kg	DMI, kg	Digestibility ^2^, g/kg				
			DM	OM	NDF	ADF	CP
Treatment, g ADY ^1^/d							
Control, 0	38.9	1.91	652	701	369	326	782
Pro, 0.6	39.9	1.91	648	706	386	307	778
Pro, 1.2	40.0	1.77	667	703	399	375	793
Vis, 0.6	39.6	2.07	674	716	443	417	767
Vis, 1.2	40.1	1.85	662	713	417	360	795
SEM	1.76	0.074	11.4	8.9	20.8	21.1	18.4
*p*-value							
Pro vs. Con	0.62	0.46	0.67	0.54	0.4	0.54	0.93
Vis vs. Con	0.66	0.57	0.23	0.21	0.02 ^Q^	0.02 ^Q^	0.83
Pro vs. Vis	0.93	0.12	0.31	0.42	0.07	0.03	0.71

^1^: The ADY sources included Procreatin7 (Pro, Guangxi Danbaoli Yeast Co., Ltd., Laibin, China) and Vistacell (Vis, AB Vista, Wiltshire, UK). The control treatment did not contain any ADY. ^2^: DM, dry matter; OM, organic matter; NDF, neutral detergent fiber; ADF, acid detergent fiber; CP, crude protein. ^Q^: Polynomial contrasts for dosage effects (quadratic, Q) of individual ADYs.

**Table 3:** Effects of active dry yeast (ADY) on N balance of Hu lambs (*n* = 6).

Item	N Intake, g/d	Fecal N, g/d	Fecal N to N Intake Ratio, mg/g	Urinary N, g/d	Urinary N to N Intake Ratio, mg/g	N Excretion, g/d	N Excretion to N Intake Ratio, mg/g	N Retention, g/d	N Retention to N Intake Ratio, mg/g
Treatment, g ADY ^1^/d									
Control, 0	42	10.1	241	8.09	192	18.2	434	23.7	565
Pro, 0.6	42	10.6	250	7.66	179	18.3	429	23.7	570
Pro, 1.2	38.7	9.24	232	8.34	218	17.6	453	21.2	548
Vis, 0.6	45.5	11.9	263	8.38	184	20.3	447	25.1	552
Vis, 1.2	40.8	9.62	231	7.58	186	17.2	417	23.6	582
SEM	1.64	1.15	20.7	0.874	19.8	1.63	29.5	1.22	29.4
*p*-value									
Pro vs. Con	0.46	0.84	0.92	0.39	0.33	0.69	0.82	0.56	0.82
Vis vs. Con	0.57	0.67	0.83	0.99	0.93	0.77	0.98	0.69	0.98
Pro vs. Vis	0.12	0.45	0.71	0.29	0.2	0.41	0.77	0.23	0.77

^1^: The ADY sources included Procreatin7 (Pro, Guangxi Danbaoli Yeast Co., Ltd., Laibin, China) and Vistacell (Vis, AB Vista, Wiltshire, UK). The control treatment did not contain any ADY.

**Table 4:** Effects of active dry yeast (ADY) on rumen fermentation parameters of Hu lambs (*n* = 6).

Item	TVFA, m*M*	pH	Molar Percentage of Individual VFA, %				Ac/Pr ^3^
			Acetate	Propionate	n-Butyrate	Others ^2^	
Treatment, g ADY ^1^/d							
Control, 0	82.9	7.13	63.2	19.4	12	5.27	3.43
Pro, 0.6	91.0	7.07	63.8	22	9.01	5.03	3.06
Pro, 1.2	89.3	7.22	63.9	21.8	10.4	3.75	2.98
Vis, 0.6	93.4	7.07	61.7	23.8	9.62	5.05	2.67
Vis, 1.2	109.4	6.96	62.5	24.8	8.29	4.26	2.66
SEM	8.43	0.090	1.45	2.23	1.686	0.402	0.248
*p*-value							
T	0.27	0.38	0.11	0.04	0.16	0.07	0.15
Sampling time	<0.001	<0.001	<0.001	<0.001	0.008	<0.001	<0.001
T × Sampling time	0.28	0.18	0.57	0.23	0.24	0.39	0.78
Pro vs. Con	0.49	0.87	0.87	0.09	0.06	0.007 ^L^	0.20
Vis vs. Con	0.08	0.32	0.08	0.006 ^L^	0.02 ^L^	0.04 ^L^	0.01 ^Q^
Pro vs. Vis	0.19	0.16	0.02	0.12	0.48	0.50	0.13

^1^: The ADY sources included Procreatin7 (Pro, Guangxi Danbaoli Yeast Co., Ltd., Laibin, China) and Vistacell (Vis, AB Vista, Wiltshire, UK). The control treatment did not contain any ADY. ^2^: Others include n-valerate, iso-butyrate, and iso-valerate. ^3^: Ac/Pr, acetate to propionate ratio. ^L,Q^: Polynomial contrasts for dosage effects (linear, L; quadratic, Q) of individual ADYs.

**Table 5:** Effects of active dry yeast (ADY) supplementation on rumen bacterial diversities of Hu lambs (*n* = 6).

Item	Observed ZOUT	PD_Whole_Tree	Pielou
Treatment, g ADY ^1^/d			
Control, 0	1194	24.6	0.702
Pro, 0.6	1109	23.2	0.653
Pro, 1.2	1263	25.4	0.666
Vis, 0.6	1142	24.6	0.661
Vis, 1.2	1111	22.5	0.643
SEM	82.3	1.31	0.0168
*p*-value			
Pro vs. Con	0.93	0.83	0.04 ^Q^
Vis vs. Con	0.50	0.50	0.02 ^L^
Pro vs. Vis	0.47	0.57	0.67

^1^: The ADY sources included Procreatin7 (Pro, Guangxi Danbaoli Yeast Co., Ltd., Laibin, China) and Vistacell (Vis, AB Vista, Wiltshire, UK). The control treatment did not contain any ADY. ^L,Q^: Polynomial contrasts for dosage effects (linear, L; quadratic, Q) of individual ADYs.

**Table 6:** Effects of active dry yeast (ADY) supplementation on relative abundance of top 20 rumen bacterial genera of Hu lambs (*n* = 6).

Genus	Treatment, g ADY ^1^/d					SEM	*p*-Value		
	Control, 0	Pro, 0.6	Pro, 1.2	Vis, 0.6	Vis, 1.2		Pro vs. Con	Vis vs. Con	Pro vs. Vis
*Prevotella*	49.3	52.8	45.8	48.6	39.7	4.34	0.99	0.34	0.24
Succinivibrionaceae_UCG-001	4.19	10.8	9.62	4.56	28.6	4.75	0.30	0.04 ^L,Q^	0.19
*Succinivibrio*	5.74	6.51	11.4	3.49	5.95	3.236	0.42	0.79	0.20
Prevotellaceae_UCG-001	5.68	4.53	4.17	4.93	0.95	1.707	0.53	0.19	0.40
*Selenomonas*	4.22	3.83	1.78	7.37	1.27	1.958	0.56	0.96	0.44
Prevotellaceae_unclassified	2.58	1.97	1.82	2.53	2.64	0.395	0.16	0.99	0.18
Rikenellaceae_RC9	2.12	1.55	1.87	1.48	1.02	0.572	0.56	0.22	0.43
Selenomonadaceae_unclassified	1.40	0.22	0.55	5.17	0.28	0.952	0.39	0.05 ^Q^	0.01
*Succiniclasticum*	2.10	0.63	1.62	2.06	0.69	0.494	0.12	0.24	0.62
*Fibrobacter*	1.48	1.04	1.56	1.00	0.76	0.335	0.20	0.26	0.37
Muribaculaceae_ge	1.29	0.80	2.01	1.04	0.56	0.519	0.86	0.44	0.25
Prevotellaceae_YAB2003	1.01	0.83	0.99	1.76	0.64	0.527	0.88	0.77	0.59
Clostridia_UCG-014	0.73	1.05	0.83	1.09	1.34	0.235	0.46	0.10	0.26
*Ruminococcus*	1.02	0.82	1.08	1.09	0.61	0.332	0.85	0.67	0.77
F082_ge	1.50	0.74	1.20	0.83	0.15	0.308	0.17	0.01 ^L^	0.10
Lachnospiraceae_unclassified	0.44	0.47	0.45	1.55	0.75	0.363	0.95	0.11	0.07
Prevotellaceae_UCG-003	1.20	0.72	0.63	0.45	0.14	0.302	0.17	0.02 ^L^	0.21
*Oribacterium*	0.48	0.48	0.47	0.63	1.05	0.184	0.93	0.16	0.13
*Treponema*	1.04	0.65	0.42	0.41	0.25	0.275	0.14	0.04 ^L^	0.39
Lachnospiraceae_ND3007	1.30	0.22	0.51	0.33	0.04	0.329	0.02 ^Q^	0.01 ^L^	0.58

^1^: The ADY sources included Procreatin7 (Pro, Guangxi Danbaoli Yeast Co., Ltd., Laibin, China) and Vistacell (Vis, AB Vista, Wiltshire, UK). The control treatment did not contain any ADY. ^L,Q^: Polynomial contrasts for dosage effects (linear, L; quadratic, Q) of individual ADYs were run for each ADY separately plus the control.

## Data Availability

The raw sequence reads of the bacterial amplicons are available in the Genome Sequence Archive of China National Center for Bioinformation under accession NO. PRJCA016785 (URL: https://www.cncb.ac.cn/?lang=en, accessed on 5 May 2023).
